# Dorsal hippocampal *N*-methyl-d-aspartate receptors underlie spatial working memory performance during non-matching to place testing on the T-maze

**DOI:** 10.1016/j.bbr.2007.07.021

**Published:** 2008-01-10

**Authors:** Stephen B. McHugh, Burkhard Niewoehner, J.N.P. Rawlins, David M. Bannerman

**Affiliations:** Department of Experimental Psychology, University of Oxford, South Parks Road, Oxford OX1 3UD, UK

**Keywords:** Hippocampus, Dorsal, Ventral, Spatial memory, Muscimol, AP5

## Abstract

Previous lesion studies have suggested a functional dissociation along the septotemporal axis of the hippocampus. Whereas the dorsal hippocampus has been implicated in spatial memory processes, the ventral hippocampus may play a role in anxiety. However, these lesion studies are potentially confounded by demyelination of fibres passing through the lesion site, and the possibility of secondary, downstream changes in associated brain structures as a consequence of their chronic denervation following the lesion. In the present study, we have used the microinfusion of muscimol to temporarily inactivate either the dorsal or ventral hippocampus in order to re-examine the contribution of the hippocampal sub-regions to spatial memory. Microinfusion studies spare fibres of passage and offer fewer opportunities for compensatory changes because the effects are transient and short-lasting. Rats were infused prior to spatial working memory testing on a non-matching to place T-maze alternation task. Spatial working memory was impaired by dorsal but not ventral hippocampal inactivation. In a second experiment, infusion of the NMDAR antagonist, D-AP5, into dorsal hippocampus also impaired spatial working memory performance, suggesting that NMDAR function within the dorsal hippocampus makes an essential contribution to this aspect of hippocampal information processing.

## 1 Introduction

A role for the hippocampus in certain kinds of memory is well established. In rodents, the hippocampus has been particularly associated with spatial learning [1]. Complete hippocampal lesions have repeatedly been shown to produce robust and lasting impairments on spatial memory tasks [2,3]. More recently, however, these spatial memory functions have been ascribed specifically to the dorsal (or septal) portion of the rat hippocampus [4,5]. For example, selective cytotoxic lesions restricted to dorsal hippocampus produce spatial learning impairments on tasks like the Morris watermaze [6, 7, see also 8]. In contrast, lesions of the ventral hippocampus are without effect on spatial memory tasks, although conversely, they, but not dorsal hippocampal lesions, are associated with reduced anxiety [9–11]. This double dissociation suggests that different hippocampal sub-regions may mediate different aspects of hippocampal function [5].

Thus far, the conclusion that spatial learning is primarily a function of dorsal hippocampus has largely been based on the results of such cytotoxic lesion studies. However, this approach is potentially confounded in that any behavioural sequelae may be the result of indirect effects of the lesion procedure on other brain areas, rather than as a direct consequence of cell loss in the target region. For example, although the cytotoxic lesion approach is a clear improvement over more traditional techniques (e.g. aspiration, electrolytic or radiofrequency-generated lesions), in terms of sparing fibres of passage [12], there nevertheless remains some evidence of demyelination of nerve fibres passing through the lesion site even with this approach [13,14]. Furthermore, it is also possible that any behavioural effects of the lesion could reflect a secondary, downstream change in an associated brain structure as a consequence of the chronic denervation of that structure following the lesion [15]. It is therefore important to re-examine the dorsal/ventral dissociation using alternative experimental approaches. For example, it is possible to produce a temporary, reversible inactivation of a specific brain region by microinfusing the GABA_A_ agonist muscimol [16], which avoids many of the potential confounds associated with the lesion approach. Microinfusion studies offer fewer opportunities than lesion studies for compensatory changes because the effects of the manipulation are transient and short-lasting. They also permit within-subjects comparisons. Somewhat surprisingly, the effects of dorsal and ventral hippocampal inactivation on spatial working memory performance have never been fully assessed.

In the present study, we therefore compared the effects of dorsal and ventral hippocampal infusions of muscimol on spatial learning using a spatial working memory, non-matching to position (discrete trial, rewarded alternation) paradigm on the elevated T-maze [17]. This task is especially sensitive to hippocampal dysfunction (see effect sizes in [7], p. 1184), and is dramatically impaired by dorsal but not ventral hippocampal lesions [7,9,18]. We therefore compared dose-response functions for muscimol infusions into both hippocampal sub-regions (Experiment 1).

In a second experiment, the importance of *N*-methyl-d-aspartate receptor (NMDAR) function, specifically in the dorsal hippocampal subregion, for spatial working memory performance was assessed. The role of NMDARs in spatial working memory is well established [19,20]. Intracerebroventricular (i.c.v.) infusion of the NMDAR antagonist, d(−)-2-amino-5-phosphonopentanoic acid (AP5) resulted in a delay-related impairment in choice accuracy on the same spatial, non-matching to place T-maze task [21]. However, the i.c.v. route of drug delivery results in NMDAR blockade in numerous brain regions, and not just the hippocampus [22]. In view of the proposal that it is the dorsal subregion of the hippocampus that is crucial for spatial working memory performance, we therefore also examined the effects on spatial non-matching to position of direct infusions of AP5 specifically into dorsal hippocampus (Experiment 2).

## 2 Methods

### 2.1 Subjects

Thirty male Lister-hooded rats (Harlan Olac, Bicester, UK; 250–363 g at the time of surgery) took part in Experiment 1. Nine male Lister-hooded rats (270–370 g at the time of surgery) took part in Experiment 2. All rats were experimentally naïve and maintained on a 12 h light/dark cycle (lights on at 7:00 a.m., with testing during the light phase). During the experiment, the animals were maintained at ∼85% of their free-feeding weight but had access to water *ad libitum*. Rats were housed in groups of 2–3 prior to surgery but were singly housed after cannulae implantation. The experiments were conducted in accordance with the United Kingdom Animals Scientific Procedures Act (1986); under project license number PPL 30/1505.

### 2.2 Apparatus

In Experiment 1, rewarded alternation took place on an elevated (∼1 m above the floor), low-walled wooden T-maze consisting of a start arm (80 cm long; 10 cm wide) joined to two identical goal arms (60 cm × 10 cm), with each arm surrounded by a 1 cm ridge. In Experiment 2, rewarded alternation took place on an elevated Y-maze (∼80 cm above the floor). Each of the three arms (50 cm × 9 cm) was surrounded by a 0.5 cm ridge and extended from a hexagonal central platform (14 cm diameter). The T- and Y-mazes contained stainless-steel food wells at the far end of each goal arm and each maze was located in a well-lit testing room containing prominent extra-maze cues such as wall posters.

### 2.3 Procedure

In both experiments, rats were trained pre-operatively. Habituation to the maze took place over a five-day period. For the first three days rats were placed on the maze in pairs and left to explore and collect food rewards for 10 min. During days four and five, the rats were placed individually on the maze and allowed to explore freely for five minutes. By this stage all the rats ate from the food wells at the ends of the arms. Training on the rewarded alternation task then followed.

Each trial in rewarded alternation had two runs: in the first, one of the goal arms was blocked, allowing the rat to enter only the other goal arm, whereupon it received 1 food pellet (45 mg Rodent Diet Formula A/I, Noyes, Lancaster, NH). During the second run, the block was removed and the rat was placed on the maze and given a free choice of either arm. Rats received two pellets for choosing the previously unvisited arm (i.e. for alternating). Choosing the arm previously visited in the sample run yielded no reward. The time between the sample and the choice runs was approximately 10 s (Experiments 1 and 2a) or 30 s (Experiment 2b). Left/right allocations for the sample and choice runs were pseudo-randomised over ten trials per day, with no more than three consecutive sample runs to the same side. The inter-trial interval was ∼3–4 min. Prior to surgery, each rat was trained on the task until they achieved a criterion of at least 80% correct alternation over two consecutive days.

In Experiment 1, allocation to surgery groups (dorsal or ventral cannulation) was based on pre-operative performance. Rats were anaesthetised with Avertin (0.29 g/kg; i.p.) and placed in a stereotaxic frame with the head level between bregma and lambda. An incision of the scalp was made along the midline, followed by a craniotomy. Stainless-steel guide cannulae (23-gauge, 12 mm) were implanted bilaterally into either the dorsal (*n* = 14; AP: −3.6 (from bregma); M–L: ±2.8 (from bregma); D–V: −0.8 (from brain surface)) or ventral HPC (*n* = 16; AP: −5.2 (from bregma); M–L: ±4.9 (from bregma); D–V: −3.6 (from brain surface)). In Experiment 2, cannulae were only implanted into the dorsal HPC (*n* = 9; coordinates as above). Cannulae were secured with three skull screws and acrylic dental cement. Microinjectors (31-gauge, 14 mm) were 2 mm longer than the guide cannulae giving a targeted D–V coordinate (from brain surface) of either −2.8 mm (dorsal) or −5.6 mm (ventral). Stylets were inserted into the guide cannulae to prevent infection and blockages.

Rats were allowed a minimum of five days post-surgical recovery after which they received training in the absence of any infusions. Rats failing to score 80% correct alternation on these sessions were given additional training.

### 2.4 Drugs

In Experiment 1, muscimol (C_4_H_6_N_2_O_2_(1/2 H_2_O); Tocris, Bristol, UK) was initially dissolved in normal saline (0.9% NaCl) at a concentration of 1 mg/ml. This stock was then diluted to create individual aliquots at each of four concentrations (0.6 mg/ml, 0.3 mg/ml, 0.15 mg/ml, and 0.075 mg/ml), which were then frozen. Saline served as the vehicle. In Experiment 2, muscimol (0.3 mg/ml) and AP5 (d(−)-2-amino-5-phosphonopentanoic acid; 5.9 mg/ml) were dissolved in PBS (pH 7.4), which served as vehicle.

On infusion days, the aliquots were defrosted and used to back-fill the microinjectors, which were connected via polyethylene tubing to a pair of Hamilton syringes (10 μl) driven by a microsyringe pump (SP250i, World Precision Instruments, England). Each rat was restrained firmly in a towel, the stylets were removed, and the microinjectors inserted through the guide cannulae into the hippocampus. Drug or vehicle (0.5 μl per side) was infused into the dorsal or ventral hippocampus at a rate of 1 μl/min (i.e. for 30 s), and the microinjectors were kept in place for an additional 60 s to allow the drug to diffuse. Infusions into each hemisphere were made simultaneously and were given 15 min prior to testing. In Experiment 1, rats received five infusions in total (saline and four doses of muscimol: 0.3 μg/side, 0.15 μg/side, 0.075 μg/side, 0.0375 μg/side). In Experiment 2a, rats received three infusions (PBS, AP5: 2.95 μg/side, muscimol: 0.15 μg/side) and in Experiment 2b, the same rats received a further 4 infusions (PBS × 2, AP5: 2.95 μg/side × 2), making seven infusions in total. Infusions were given in a counterbalanced, pseudo-random order with at least 48 h between each infusion. In addition, performance was assessed again 24 h after each infusion and rats failing to score 80% or more on these drug-free sessions received extra training before the next infusion was administered.

### 2.5 Histology

On completion of the experiment, rats were perfused transcardially and their brains were removed, sectioned, and stained with cresyl violet for verification of the injection sites.

## 3 Results

### 3.1 Experiment 1

**Fig. 1:**
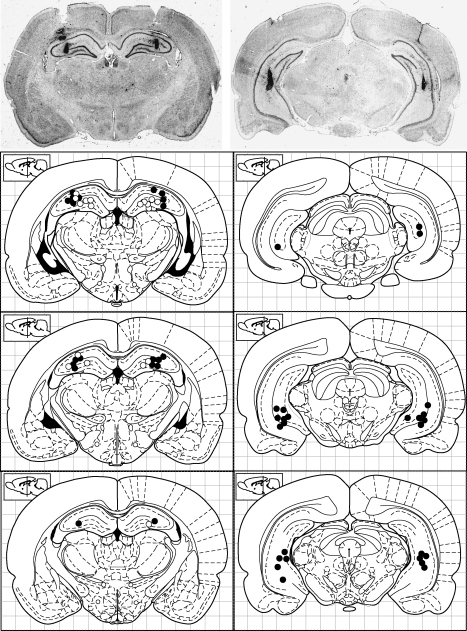
Reconstructions and photomicrographs of microinjection sites in dorsal (left panel) and ventral hippocampus (right panel) for experiment 1 (black circles) and experiment 2 (white circles). The anterior-posterior coordinates (from bregma) for the dorsal sections are from top-to-bottom: −3.80, −3.60, −3.30; and for the ventral sections: −6.30, −6.04, −5.60 according to the atlas of Paxinos and Watson [36].

In Experiment 1, of the 30 rats that began the experiment, 23 rats completed all testing and had accurate bilateral cannulae placements and were included in the final analyses (10 dorsal, 13 ventral; Fig. 1). The intra-hippocampal implantation of guide cannulae did not result in any post-surgical deficits in rewarded alternation in either dorsal (mean score = 94%) or ventral (mean score = 90%) cannulated rats. Indeed post-operative performance was slightly better than pre-operative performance in both groups. Student's *t*-tests carried out for each group confirmed that there were no differences between pre-operative and (uninfused) post-operative performance (dorsal: *t*(9) = −0.483; *p* > 0.2; ventral: *t*(12) = −1.15; *p* > 0.2).

**Fig. 2:**
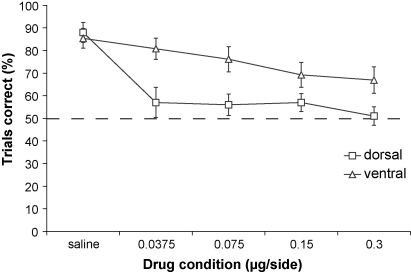
The effects of muscimol (0.0375, 0.075, 0.15, 0.3 μg/side) microinfusion into the dorsal and ventral hippocampus on rewarded alternation performance on the T-maze. Data points represent mean percentage correct trials (±S.E.M.) The broken line shows chance performance (dorsal group, *n* *=* 10; ventral group, *n* *=* 13).

The amount of alternation following saline infusion into either dorsal or ventral hippocampus was similar and comparable to uninfused performance (dorsal = 88%; ventral = 85%). In contrast, bilateral infusion of muscimol into the dorsal hippocampus produced a decrease in alternation levels (see Fig. 2). These data were analyzed using a repeated-measures ANOVA [model: cannula placement_2_ × (drug treatment_5_ × *S*_23_)]. The ANOVA revealed a significant effect of cannula placement (*F*_1,21_ = 14.44; *p* < 0.001) and drug treatment (*F*_4,84_ = 9.01; *p* < 0.001) but the interaction was not quite significant (*F*_4,84_ = 2.05; *p* = 0.09).

Analysis of simple main effects and subsequent pairwise comparisons (using Sidak's method, see [23]) found an effect of cannula placement at three doses of muscimol: 0.0375 μg/side (*F*_1,21_ = 8.94; *p* < 0.01), 0.075 μg/side (*F*_1,21_ = 6.94; *p* < 0.05) and 0.3 μg/side (*F*_1,21_ = 4.45; *p* < 0.05) with the dorsal group exhibiting greater impairment than the ventral group. The groups did not differ following saline infusion (*p* > 0.2) or when the muscimol dose was 0.15 μg/side (*p* > 0.1). Simple main effects analysis also revealed an effect of drug within the dorsal cannula placement group (*F*_4,18_ = 6.86; *p* < 0.005), with all doses of muscimol leading to performance impairment compared to saline infusion. In contrast, there was no effect of drug within the ventral placement group (*F*_4,18_ = 2.7; NS). In other words, although there was some suggestion that performance was decreasing as the dose of muscimol increased (see Fig. 2), no individual dose of muscimol into the ventral hippocampus significantly impaired performance compared to saline infusion.

On alternate days, the rats received 10 trials of rewarded alternation in the absence of any infusions to investigate the possibility of permanent or progressive damage to the targeted regions. Importantly, this data showed that rats from both groups alternated on more than 85% of trials 24 h after receiving infusions. Post-infusion performance of dorsal and ventral cannulated rats was investigated with a repeated-measures ANOVA [model: cannula placement_2_ × (block_5_ × *S*_23_)]. The ANOVA found no effect of cannula placement (*F*_1,21_ = 2.37; NS), or block (*F*_4,84_ = 0.49; NS), and there was no interaction (*F*_4,84_ = 1.99; NS). This result demonstrates that the T-maze impairments identified in the previous analysis were reversible and only evident while the drug was actually present in the hippocampus.

### 3.2 Experiment 2

In Experiment 2, all nine rats had accurate bilateral dorsal hippocampal cannulae placements (Fig. 1). Experiment 2 was divided into two stages. Procedurally, Experiment 2a was identical to Experiment 1. Experiment 2b was identical to Experiment 2a except that a 30 s delay was interposed between the sample and choice runs of each trial of rewarded alternation (see [21]).

In Experiment 2a, the amount of alternation following PBS infusion into the dorsal hippocampus was similar and comparable to uninfused performance (uninfused = 90%, PBS = 83%). In contrast, there was a substantial drop in choice accuracy following the infusion of either muscimol (58%) or AP5 (67%). A one-way repeated-measures ANOVA [model: drug condition_2_ × *S*_9_], found a main effect of drug condition (*F*_2,16_ = 6.3; *p* < 0.01) and post hoc pairwise comparisons (Student's Newman–Keuls) revealed significant differences between the vehicle and both drug conditions (*p* < 0.05) but not between the two drug conditions (*p* > 0.2).

**Fig. 3:**
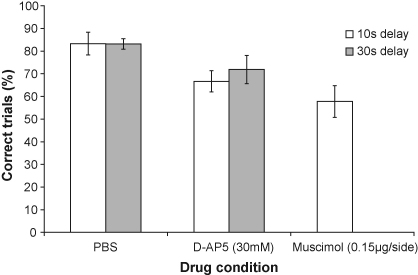
The effects of D-AP5 (30 mM, 2.95 μg/side) and muscimol (0.15 μg/side) microinfusions into the dorsal hippocampus on rewarded alternation performance on the T-maze without delay (white bars) or with 30 s delay (grey bars). Columns represent mean percentage correct trials (±S.E.M.) (Dorsal group, *n* *=* 9).

The introduction of the 30 s delay in Experiment 2b did not lead to a further drop of performance in either infusion condition (PBS = 83%; AP5 = 72%). Although not strictly appropriate (since the data were not derived in a counterbalanced manner), a repeated measures ANOVA was performed on the combined data of Experiments 2a and 2b (see Fig. 3). The ANOVA revealed a main effect of drug condition (*F*_1,7_ = 11.75; *p* < 0.05) but no effect of delay or drug condition × delay interaction (both *F* < 1; NS).

Finally, performance accuracy (90%) during drug-free testing carried out after the last infusion confirmed that there was no lasting damage due to cannulae implantation or the microinjection process.

## 4 Discussion

Experiment 1 showed that selective inactivation of the dorsal hippocampus results in significant impairment of spatial working memory performance whereas equivalent inactivation of ventral hippocampus does not. This result is consistent with previous lesions studies [7,18], but advances on these earlier findings by demonstrating that such dorsal/ventral differences are not due to indirect effects such as demyelination or compensatory changes that result from permanent brain lesions. Also, the impairment resulting from infusion of AP5 directly into the dorsal subregion (Experiment 2) identifies the importance of *N*-methyl-d-aspartate receptor (NMDAR) function in the dorsal hippocampus for spatial working memory performance.

This data, and that obtained from previous cytotoxic lesions studies, are consistent with findings from a variety of different approaches which suggest a functional specialization along the septotemporal (dorsal/ventral) axis of the hippocampus. Evidence from electrophysiological single unit recording studies in both rats [24,25] and primates [26], from *c-fos* activation studies [27], and from structural magnetic resonance imaging studies [28], are all consistent with a functional dissociation between dorsal (posterior in primates) and ventral (anterior in primates) hippocampus, and with a preferential role for dorsal hippocampus in spatial learning and memory.

To our knowledge, this experiment constitutes the first demonstration of differential effects of dorsal and ventral hippocampal inactivation on spatial working memory performance using T-maze rewarded alternation. It is worth noting, however, that the effects of selective inactivation of dorsal and ventral hippocampus on delayed alternation tasks have been examined previously using operant paradigms, but with mixed results. For example, Mao and Robinson [29] trained rats to press a lever on the left or right-hand side of the front wall of the operant chamber as indicated by the presence of a light stimulus (equivalent to the sample phase in the present study), then press a lever on the rear wall before being given a free choice of either front wall lever (equivalent to the choice phase in our study). The rats were required to alternate lever presses on the front wall in order to obtain food rewards. Although this task has some similarity to our T-maze task (both require alternation behaviour), bilateral infusions of muscimol into the dorsal hippocampus did not affect the proportion of correct responses. It is worth pointing out, however, that the doses used by Mao and Robinson were lower than the doses used in the present study (0.003–0.01 μg compared to 0.0375–0.3 μg in the present study). It is also worth noting that in their study dorsal infusions of higher doses of muscimol (0.03–0.2 μg) interfered with the rats’ ability to perform various non-spatial aspects of task performance such as correct lever presses at the sample stage and the rear lever press between the choice and sample phases. In a separate study, Maruki et al. [30], trained rats to alternate between pressing the left and right lever on each trial. To prevent mediating strategies, the rat was required to make a touch response to the central food-well during the ITI. Using this continuous alternation paradigm, the authors found that infusions of muscimol (0.07 μg/side) into the dorsal hippocampus reduced correct responding when the ITI was 20 s but not when it was 3 s. In a second experiment, they reported no effects of ventral infusions of muscimol relative to saline vehicle infusions on the same task. However, inspection of the data across the two experiments shows that the levels of performance in rats receiving muscimol infusions into dorsal hippocampus (70.9% correct) and in rats receiving muscimol into ventral hippocampus (72.3% correct) were virtually indistinguishable. The present study thus provides the first clear demonstration that spatial working memory performance is sensitive to dorsal but not ventral hippocampal inactivation.

As noted by Mao and Robinson [29] direct intra-hippocampal infusion carries the risk of non-specific effects on performance. For example, it is possible that unequal quantities of muscimol entering each hemisphere could lead to a turning bias. First, it is worth pointing out that in our study both hemispheres were infused simultaneously. Second, in Experiment 1, rats receiving dorsal HPC infusions exhibited side preferences (i.e. they exclusively entered the same goal arm on the choice phase of each trial of a session) in only 13 of the 40 sessions (10 rats × 4 muscimol doses). It is also worth pointing out that such side preferences are commonly observed in rats with permanent HPC lesions and in our experience these preferences are not dependent on the size of the lesion in one particular hemisphere. They are generally considered to be a consequence, rather than a cause, of the memory impairment. Furthermore, the fact that in Experiment 1, errors were made to both goal arms in almost 70% of sessions argues against a performance account based solely on side-preferences elicited by unequal quantities of muscimol entering each hemisphere.

An alternative account of the septotemporal differences found in Experiment 1 is that the spread of the drug within the hippocampus is simply greater when infused into dorsal than ventral HPC, effectively creating a larger dorsal than ventral HPC inactivation. Without autoradiography to determine the extent of muscimol diffusion we cannot rule out this possibility completely. However, when the data from the present study are considered along with the previous cytotoxic lesion studies, such an account seems unlikely. Evidence from permanent lesion studies suggests that lesion size itself is not the critical determinant of degree of dysfunction. For example, Moser and colleagues [6] found that rats with lesions encompassing 20–40% of HPC tissue starting at the ventral pole showed normal spatial learning in the watermaze, whereas rats with lesions of 20–40% of HPC tissue starting at the dorsal pole were profoundly impaired. Furthermore, we have previously tested rats with dorsal and ventral cytotoxic lesions of very similar sizes (50–55%) on the same T-maze rewarded alternation task as used in the present study, and have found that whereas dorsal lesioned animals resemble animals with complete hippocampal lesions, exhibiting chance levels of performance, ventral lesioned animals perform as well as sham operated controls [9]. These data strongly suggest that lesion location, and not lesion size, is the critical factor.

In the present study, although no dose of muscimol into the ventral HPC significantly impaired performance compared to vehicle, there was some suggestion of a dose related decline in performance (see Fig. 2). This could reflect a role, albeit more limited, for the ventral hippocampus in spatial information processing. However, it is also possible that this slight drop in performance may reflect the spread of muscimol into mid-hippocampal and/or dorsal regions. In this regard, it is worth re-iterating that ventral hippocampal lesions, removing approximately 50% of the structure starting from the temporal pole, had absolutely no effect on performance on this very same spatial working memory, rewarded alternation task [7,9] Therefore, the evidence suggests that spatial working memory tasks such as rewarded alternation on the T-maze, like spatial reference memory tasks such as the standard fixed location version of the watermaze, are sensitive to dorsal but not equivalently sized ventral HPC lesions or inactivations.

Nevertheless, it may be unwise to completely exclude a role for the ventral HPC in spatial information processing. Single-unit studies have shown that cells selective for specific spatial locations (“place cells”) do exist in ventral HPC, albeit they are fewer in number and their place fields are larger than those in dorsal HPC. Although the electrophysiological data are limited, McNaughton and colleagues have suggested a septotemporal gradient in spatial selectivity with higher resolution spatial discriminations processed in dorsal HPC [24,25]. In this way, cells in dorsal HPC might code for specific locations within a particular environment whereas ventral cells might code for different environments or contexts, although this may be just a subset of the contextual information that can be processed by the ventral subregion [5]. It remains to be seen if tasks requiring discrimination between different spatial environments or contexts are more sensitive to lesions of the ventral HPC.

The results of Experiment 2 show that NMDARs in dorsal hippocampus contribute to performance on spatial working memory tasks. Previous work in this laboratory showed that i.c.v. infusion of AP5 impaired performance on the same rewarded alternation task [21]. The present results now suggest that this effect is, at least in part, due to the blockade of NMDARs in dorsal hippocampus. Similar spatial working memory impairments have also been observed following AP5 infusions into dorsal hippocampus on a spatial, matching-to-position version of the watermaze task [19]. Both of these results are consistent with the hypothesis that NMDAR-mediated forms of synaptic plasticity, similar to experimentally induced long-term potentiation [LTP; 31, 32], may underlie a flexible, rapidly acquired form of spatial memory, as typified by these spatial working memory tasks [20,33].

The observation that introducing a further 20 s delay failed to exacerbate the spatial working memory deficit might be considered surprising. In contrast to the present results, Tonkiss and Rawlins [21] showed that increasing the delay by 20 s did in fact increase the impairment in rats infused with AP5 i.c.v., on the same T-maze spatial working memory task. However, it is worth pointing out that in both cases, the effect of increasing delay was not investigated as part of a fully counterbalanced design, and therefore some caution is required when interpreting these results. Steele and Morris [19] did report a delay-dependent impairment in their matching to position watermaze task with a fully counterbalanced design, although it is worth noting that performance at the shortest delay was not consistently spared in their study.

We have also recently tested genetically modified mice in which the NR1 subunit of the NMDAR has been selectively deleted, specifically from the dentate gyrus subfield of the hippocampus [34]. These mice exhibit deficits in LTP in both the medial and lateral aspects of the perforant path, whereas LTP in the CA1 subfield is normal. We assessed spatial memory on the radial maze in these mice, and found that they exhibited a very specific spatial working memory deficit. As with the present study, the animals displayed a spatial working memory deficit at the shortest-possible delays that we were able to test. It is of course possible that at even shorter delays performance may be spared in the AP5 rats and in the dentate gyrus NR1 knockout mice. Equally, it could be that by blocking NMDAR-dependent synaptic plasticity in the hippocampus, we have disrupted an existing spatial representation of the environment, or prevented the expression of memory in some way, for example, by impairing the ability to select the appropriate spatial response on the basis of information retrieved from memory (both of which might be expected to result in a delay-independent deficit). Against this, the dentate gyrus-specific NR1 knockout mice displayed perfectly normal acquisition and performance on the reference memory component of the radial maze task (i.e. learning which 3 arms were baited and which three arms were never baited). It may therefore be that disrupting hippocampal synaptic plasticity prevents the animals from rapidly forming a short-term spatial representation of the maze arm visited on the sample run. This ability to record or represent the recent experience of a particular spatial location or stimuli (i.e. the maze arm) is likely to be of crucial importance for efficient spatial working memory performance [35].

In conclusion, the present research has confirmed and extended previous findings from lesion studies, namely that the dorsal hippocampus has a greater involvement in spatial working memory than the ventral hippocampus. Furthermore, NMDAR activation within the dorsal hippocampus makes an essential contribution to this aspect of hippocampal information processing.
